# First Restoration Experiment for *Gongolaria barbata* in Slovenian Coastal Waters. What Can Go Wrong?

**DOI:** 10.3390/plants10020239

**Published:** 2021-01-26

**Authors:** Martina Orlando-Bonaca, Valentina Pitacco, Petra Slavinec, Milijan Šiško, Tihomir Makovec, Annalisa Falace

**Affiliations:** 1 Marine Biology Station Piran, National Institute of Biology, Fornače 41, SI-6330 Piran, Slovenia; Valentina.Pitacco@nib.si (V.P.); sslavinecpetra@gmail.com (P.S.); Milijan.Sisko@nib.si (M.Š.); Tihomir.Makovec@nib.si (T.M.); 2 Department of Life Sciences, University of Trieste, Via L. Giorgieri 10, 34127 Trieste, Italy; falace@units.it

**Keywords:** brown algal forests, *Cystoseira*, *Gongolaria barbata*, restoration, ex situ cultivation, herbivorous fish, northern Adriatic Sea

## Abstract

The global decline of brown algal forests along rocky coasts is causing an exceptional biodiversity loss. Regardless of conservation efforts, different techniques have been developed for large-scale restoration strategies in the Mediterranean Sea. In this study we tested ex situ pilot restoration of *Gongolaria barbata* (=*Treptacantha barbata*) for the first time in Slovenian coastal waters. Healthy apical fronds of the species were collected and the development of recruits on clay tiles was followed under laboratory conditions for 20 days. Despite the experimental difficulties experienced, especially due to the lack of antibiotics to prevent the growth of the biofilm, *G. barbata* recruits were outplanted in the sea on two concrete plates with 48 tiles each, protected by purpose-built cages to avoid grazing by herbivorous fish. The high survival rate of juveniles after four months in the field (89% of the tiles on the plate that was constantly protected) suggests that outplanting *G. barbata* is an operable approach for restoration efforts in the northern Adriatic Sea. Our first experiment in Slovenian coastal waters provides new information for the optimization of the best practices during the laboratory cultivation and addresses the early steps of restoration and introduction of young thalli in the natural environment.

## 1. Introduction

Among perennial Mediterranean canopy-forming macroalgae, erect brown *Cystoseira sensu lato* species [1], henceforth referred to as *Cystoseira* (Fucales, Phaeophyceae), play an important role as habitat-builders on rocky bottoms [2,3,4]. They form the so-called brown algal forests that for decades have been considered one of the most productive assemblages in the Mediterranean Sea [5,6]. The three-dimensional structure of such habitats provides food sources, substrata for settlement and shelter for many smaller algae, invertebrates, and fish [7,8,9,10]. Moreover, *Cystoseira* spp. produce several potentially bioactive metabolites, i.e., fatty acids, steroids, phlorotannins, polysaccharides, and terpenoids, which have diverse benefits for humans [11]. Indeed, antiviral, antibacterial, antioxidant, anti-inflammatory, and antifungal activities have been confirmed for numerous *Cystoseira* species [12,13,14].

With the exception of *Cystoseira compressa* (Esper) Gerloff & Nizamuddin, all other *Cystoseira* species are included in Annex II (List of endangered or threatened marine species in the Mediterranean) of the Barcelona Protocol concerning Specially Protected Areas and Biological Diversity [15] and in Annex I of the Bern Convention [16]. According to the Habitat Directive [17], all *Cystoseira* species are considered “Habitat of Community Interest by the EU (Habitat Reef—code 1170 Annex I)”. These species are also under surveillance by the IUCN (International Union for the Conservation of Nature), the RAC/SPA (Regional Activity Centre for Specially Protected Areas established under the Barcelona Convention), and the MedPAN (Mediterranean network of Marine Protected Areas (MPAs)).

In the past decades, several scientists have observed and reported that coastal ecosystems are exposed to interplaying local stressors (mainly from anthropogenic sources), which can result in shifts between alternative habitats [18,19,20,21]. Since *Cystoseira* follow long-term periodicity, its disappearance from shallow rocky bottoms is considered as indicative of severe environmental degradation [22,23,24,25]. Under significant anthropogenic stressors, *Cystoseira* is replaced by smaller and less complex turf-forming algae [26]. These low-lying algae then form a substitute stable state on the bottom that seems to inhibit recolonization by canopy-forming species [27,28]. According to Strain et al. [29], in order to prevent shifts from canopy to turf-forming algae, priority should be given to the management of nutrient levels, especially in enclosed bays and estuaries, since it has been demonstrated that Fucoids are stronger competitors for space than algal turfs in oligotrophic water conditions [23,30,31].

The eastern Mediterranean is known as one of the most altered marine regions, exposed to water warming (climate change), invasion by thermophilic species, and overfishing, all of which cause many negative changes, including the decrease of brown algal forests [32], and the dominance of turf-forming taxa and non-indigenous macroalgae [33]. Moreover, anthropogenic climate change [34] is causing unprecedented alterations in marine ecosystems and is known to induce species re-distributions, i.e., it is expected that under a warmer climate, species will move towards higher latitudes, higher altitudes, or deeper waters [35]. However, in the shallow Gulf of Trieste, the northernmost part of both the Adriatic and the Mediterranean Seas, these migrations are not possible. Some variability in macroalgal assemblages is also ascribed to the synergy between anthropogenic factors, like pollution and habitat destruction [10,18,23] and natural environmental factors, such as storms and sediment scour [18]. Moreover, overgrazing by sea urchins [36,37,38,39] and herbivorous fishes [40,41] in some Mediterranean areas is also considered one of the major threats to brown algal forests. Some *Cystoseira* species have already been driven to regional extinction [22], and despite the implementation of significant conservation actions, many degraded brown algal forests have not recovered [26,42]. Indeed, in the absence of adult thalli in a given area, their natural recovery is hindered by the very limited spread of *Cystoseira*, due to the rapid fertilization of its eggs and the sinking of zygotes [43]. These facts underlie the need for intervention actions to restore endangered habitats, and for some *Cystoseira* species methodological protocols have already been tested [43,44,45]. In this context, we consider the term “restoration” as “the process of assisting the recovery of damaged, degraded, or destroyed ecosystems” [46], which includes preventive management aimed at reducing pressures, and habitat reconstruction [47].

Three restoration techniques are available for *Cystoseira* in the Mediterranean Sea. The first technique provides the transplantation of adult thalli onto artificial modules, by fixing the holdfasts using arched hooks or by covering the basal part of the plant with polyurethane foam [48], and by fixing the thalli with epoxy glue [49]. For the second technique, bags containing fertile receptacles were tied to a pick and directly fixed near the sea bottom (in situ) at selected restoration sites [44,50]. The third technique provides the outplanting of juveniles cultured ex situ under laboratory conditions [43,44,45,51,52]. The latter two techniques are recommended for the recovery of endangered species in order to avoid the exhaustion of natural donor populations [43], especially for species with a low dispersal capacity [52,53].

In line with the Water Framework Directive [54] and Marine Strategy Framework Directive [55] requirements, macroalgae have been regularly monitored since 2006 for the evaluation of the ecological and environmental status of Slovenian coastal waters [56]. Changes in macrophyte spatial and seasonal diversity and a loss of canopy-forming taxa (especially *Cystoseria*) were detected during the last decade [57]. In the upper-infralittoral zone of Slovenian marine waters *Gongolaria barbata* (Stackhouse) Kuntze (=*Treptacantha barbata*) and *C. compressa* are still quite frequent, while other species belonging to this group are already rare. However, the abundance of the most widespread *Cystoseira s.l.* species is currently slightly declining in coastal areas, with a consequent replacement by turf-forming taxa [57]. Moreover, Savonitto et al. [45] have reported that the distribution of *G. barbata* in the Italian part of the Gulf of Trieste has already declined to the point that it is now present only in Slovenian coastal waters. The results for this geographic area confirm the global regression of canopy-forming taxa at many Mediterranean sites [2,18,21,22,58,59]. The increasing coverage of turf-forming taxa in Slovenian waters seems to be related to hydromorphological human-induced modifications of the coastline and high sediment resuspension rates [57] rather than to nutrient enrichment, since recent studies underline the oligotrophication of the northern Adriatic Sea over the last decades [60]. However, the negative effect of native herbivorous fish has also been documented for *Sargassum vulgare* C. Agardh thalli [61].

The actions planned with the framework of a research project relating to the evaluation of the status of Adriatic brown algal forests (ARRS, J1-1702) included the culture of *G. barbata* in controlled rooms, and the outplant of juveniles in the natural environment. The overall objective of the research presented in this paper was to test the feasibility of *Gongolaria barbata* ex situ restoration in Slovenian coastal waters, by evaluating the survival rate and growth of young thalli during the most critical first four months after the outplanting, with the intent to implement some sustainable restoration actions.

## 2. Materials and Methods

### 2.1. Fieldwork and Laboratory Work

The study area is located in the Gulf of Trieste, which is a shallow semi-enclosed embayment located between Cape Savudrija (Croatia) and Grado (Italy) and comprising the entire Slovenian coastline (46.7 km), with an average depth of around 21 m. The average salinity of the seawater is around 37, and is influenced by fresh water inputs near the coast, mainly from the Soča (Isonzo) River [62].

**Figure 1:**
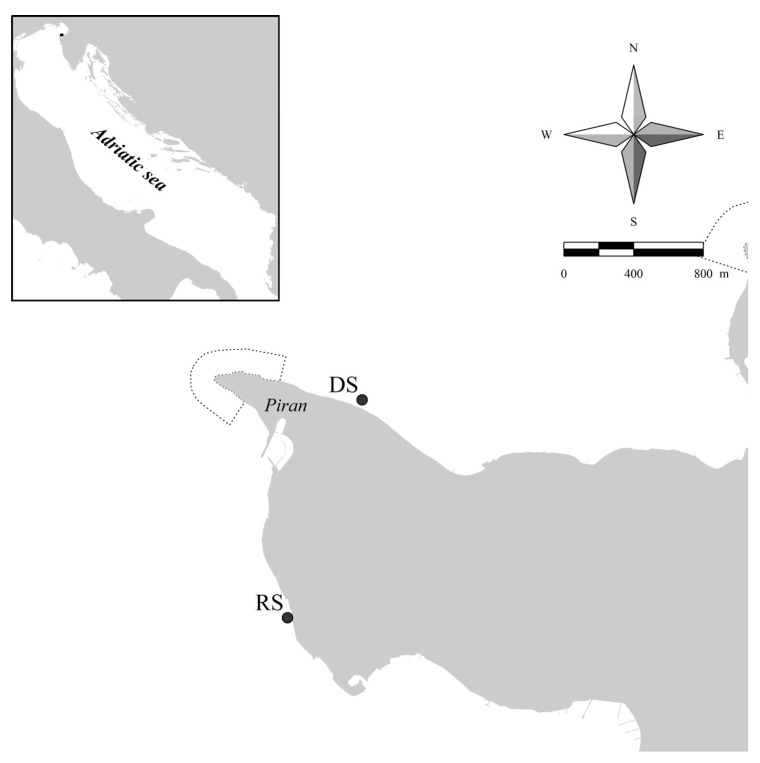
Study area in the Gulf of Trieste. DS—donor site of fertile apices of *Gongolaria barbata*, and RS—receiving site of ex situ cultivated recruits on clay tiles, in front of the Marine Biology Station Piran (Slovenia).

The donor site DS (Figure 1) for healthy apical fronds of *Gongolaria barbata* was selected according to the results of SCUBA diving surveys of *Cystoseira* assemblages, performed during the 2019 spring-summer period. At this location, *G. barbata* thalli are covering a belt approximately 100 m long, at a depth of 1 to 3 m, where the species has around 70% coverage on the rocky bottom. During the preliminary survey of the donor site (DS) on 11 March 2020 the apical fronds of *G. barbata* were already holding mature receptacles. Unfortunately, due to the global COVID emergency, it was not possible to start the planned experiment in the following six weeks. Therefore, the *G. barbata* apical fronds were collected on 23 April 2020, according to the non-destructive sampling strategy proposed by Falace et al. [43]. Healthy apical fronds of ca. 3 cm in length were collected at a depth of 1.5 to 2.5 m. No specific authorizations were necessary for the collection of the biological material, since the selected site is not included in the nearby marine protected area, i.e., the Cape Madona Nature Monument, at the far end of the Peninsula of Piran.

**Figure 2:**
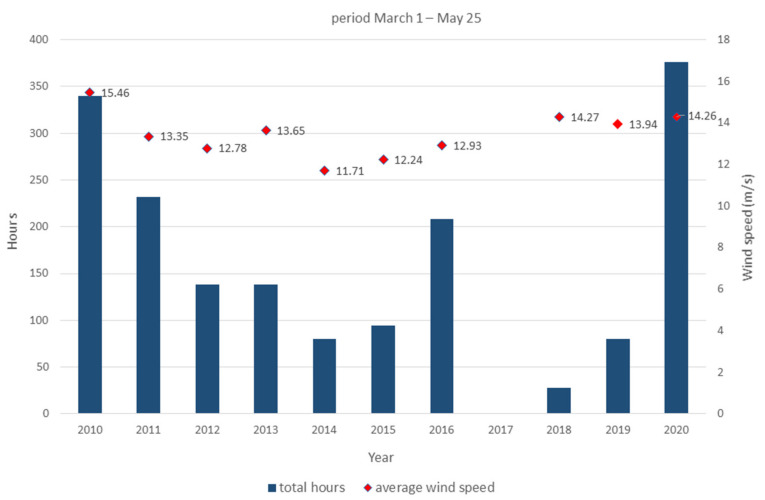
Total hours and Bora wind speed calculated for the time interval from 1 March to 25 May, in the period 2010–2020 (data from the oceanographic buoy VIDA, https://www.nib.si/mbp/en/).

**Figure 3:**
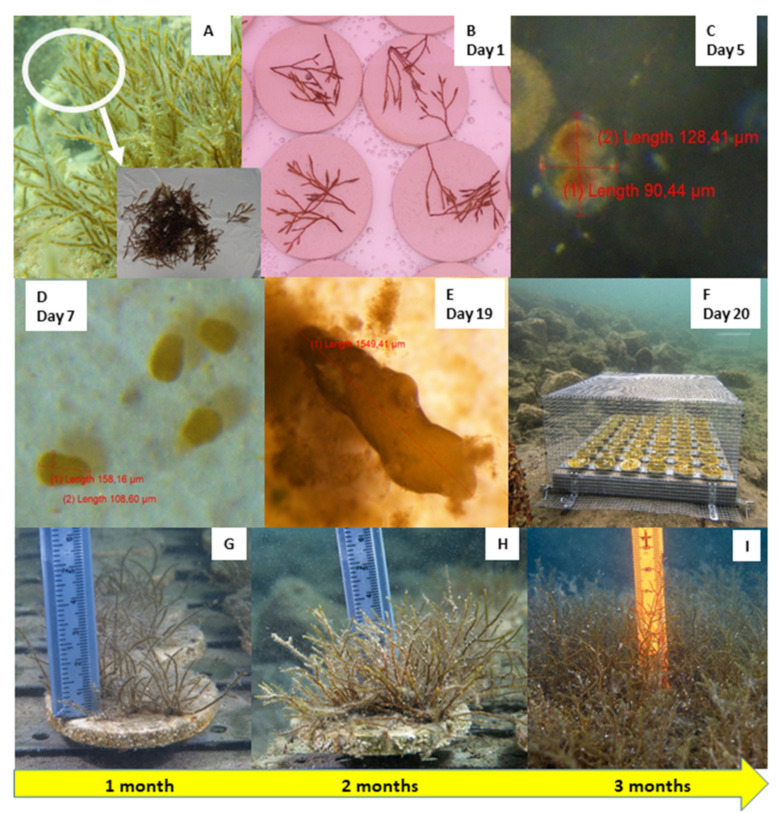
Experimental setup and zygote development in *Gongolaria barbata* recruits. (**A**) Fertile thalli and apical fronds from natural populations. (**B**) Fertile apical fronds placed on experimental rough clay tiles on day 1. (**C**) Zygotes developed on day 5 on glass slides. (**D**) Embryos adhered to the substrate of tiles on day 7. (**E**) Recruit on day 19. (**F**) Concrete plates with recruits on clay tiles placed in the sea on day 20 and protected with cages. (**G**) Recruits at 1 month in the sea. (**H**) Recruits at 2 months in the sea. (**I**) Recruits at 3 months in the sea.

After the sampling, the apices were immediately transported to the laboratory of the Marine Biology Station in Piran. The apices were examined under a stereomicroscope and it was found that there were fewer mature receptacles and that they were smaller than the month before (average length >8 mm in March, and <5 mm in April). We assumed that the largest ones were probably removed from the fronds due to strong Bora events that characterized the Gulf of Trieste in the second half of March and the beginning of April. During spring 2020, this wind blew cumulatively for many more hours than in the previous years (data from the oceanographic buoy VIDA, https://www.nib.si/mbp/en/; Figure 2). The fronds were carefully cleaned using a soft brush and rinsed with filtered seawater to remove visible biofouling and detritus (Figure 3A). Then they were wrapped in aluminium foil and left to rest for 24 h in the fridge (approx. 5 °C), since thermic shock facilitates the release of zygotes [45].

**Figure 4:**
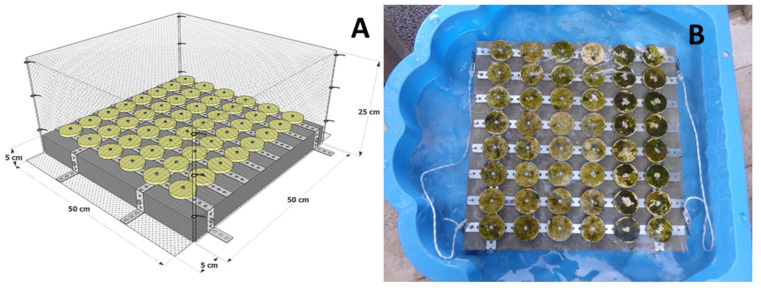
(**A**) Outplanting structure formed by a concrete plate and a protection cage for *Gongolaria barbata* recruits on clay tiles. (**B**) Two concrete plates with 48 clay tiles each, completely covered also by biofilm, were placed in the sea on day 20.

Temperature (+20 °C), photoperiod (15:9 h of light:dark cycle), and measured light intensity (125 μmol photons m^−2^ s^−1^) in the environmentally controlled room were selected according to the protocol of Falace et al. [43]. Light equipment specifications: 4 Osram Fluora Fluorescent tubes 36 W wih length 120 cm and light output of 1400 lumens per aquarium. Three experimental aquaria (volume 40 L) were prepared, rough clay tiles were placed on the bottom (diameter = 6 cm, with a 0.6 cm hole in the middle to secure the tile onto the outplanting structure, Figure 4A), and then the aquaria were filled with filtered seawater (0.22 μm pore size) to a maximum height of 1 cm in order to prevent the apices from floating. No filters were used in the aquaria. On day 1 of the experiment (Figure 3B), apices with mature receptacles were placed on all clay tiles (two aquaria with 30 and one with 48 tiles). Three additional replicates per aquarium were positioned on glass slides in order to observe and photograph zygote development with a stereomicroscope, without stressing the apices on the treatment clay tiles.

During the laboratory cultivation (see Section 3.1), Von Stosch’s enriched filtered seawater was chosen as the culture medium to accelerate the growth of *G. barbata* thalli [43,52]. Due to the COVID emergency, it was not possible to obtain the proper antibiotic on time to prevent the massive growth of bacteria [52]. On the days when the water was not changed, salinity was monitored and kept around 37 with the addition of distilled water.

### 2.2. Outplanting and Monitoring in the Field

On day 20 of the experiment, two concrete plates (for paving walking surfaces, Figure 4A) with *G. barbata* recruits on 48 clay tiles, were placed in the sea. The plates were protected by purpose-built cages (Figure 3F and Figure 4A), which were secured with metal wedges.

Photographic monitoring of the two plates with tiles in the field was performed monthly for four months (Time 1 to 4). The photos taken during each SCUBA survey were analyzed in the lab using ImageJ software [63] to assess the survival and length of the thalli. The survival rate was measure as the percentage of tiles with juveniles (checking the overall presence/absence of juveniles on each tile) in the fourth month. The length of thalli was measured for a total of 30 randomly selected specimens, for each plate and each sampling time. The cages were cleaned from epiphytes monthly in order to prevent shading.

### 2.3. Data Analysis

A two-way robust ANOVA with repeated measures based on trimmed means (20% trimming level) [64] was performed for main effect and interaction to check for differences in thalli length between two fixed factors: time (with 4 levels; T1 to T4) and plate (with 2 levels: CA and CB). The test was chosen because it is more robust to deviation from normality and homogeneity of variance than the classical two-way ANOVA. A trimmed mean discards the defined percentage at both ends of the distribution, achieving nearly the same amount of power as the mean in case of a normal distribution, and reduce substantially standard error in presence of outliers [64]. The statistical outcome measure of this test Q is interpreted in the same fashion as the traditional F statistic [64]. Tukey’s multiple comparisons of means were used to compare the two cages independently at each sampling time. Statistical analyses were performed using R 4.0.2 [65], and the following packages: ggplot2 [66] and WRS2 [64].

## 3. Results

### 3.1. Laboratory (Ex Situ) Cultivation

On days 2, 3, and 4, it was not possible to see massive zygote release on the tiles in the aquaria with the naked eye. However, a low release of zygotes was confirmed by observing the tiles on glass slides under a stereomicroscope. The first constraint was high water evaporation in the controlled room, due to the presence of fans that could not be turned off. In order to avoid the drying of the apical parts, it was necessary to add marine water diluted with distilled water on a daily basis, keeping water depth at 1 cm. The salinity value was regularly controlled and kept at around 37. On day 4, all the apices were removed from the aquaria and the water level was raised to 4 cm (around 9 L per aquarium). The first mitoses became visible on glass slides on day 5 (Figure 3C) and, on day 7, it was noted that the survived zygotes were well attached to the clay tiles (Figure 3D).

The first water change was performed on day 8 and Von Stosch’s enriched filtered seawater was added. The development of spots of bacteria on the tiles was observed on day 9. Due to the lack of antibiotics, the only possible prevention measure was continuous aeration of the aquaria using bubbling and water multifunction pumps (≈300 L h^−1^ flow) in order to increase oxygenation and hydrodynamics. In the second week, the culture medium in the aquaria was renewed every three days.

**Figure 5:**
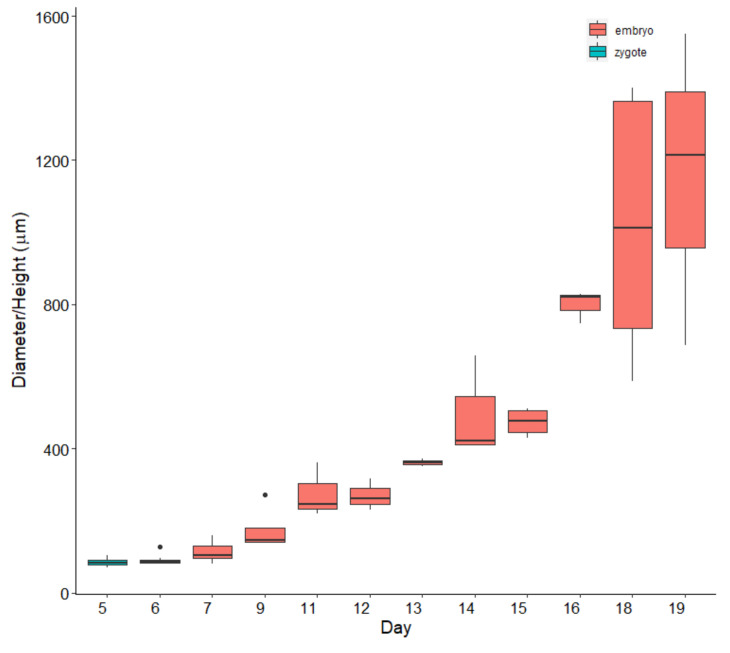
*Gongolaria barbata* zygote diameters (days 5 and 6) and embryo height (days 7 to 19) during laboratory cultivation. Coloured box represents interquartile range, central bar represents the median, vertical lines indicate the largest values within 1.5 times the interquartile range above the 75° percentile or the smallest below the 25° percentile, black points are values more than 1.5 times and less than 3 times the interquartile range.

From day 15 to day 19, the culture medium in the aquaria was renewed every day, since the tiles were almost completely covered by biofilm (Figure 4B), and the walls of the aquaria were overgrown, as well. On day 19 (Figure 3E), the average height of the measured recruits was 1.16 mm (Figure 5) and it was decided to prepare them for the outplanting project, to prevent suffocation of *G. barbata* thalli by bacteria.

### 3.2. Outplanting and Monitoring in the Field

On day 20 (13 May), two concrete plates (Figure 4B) with 48 clay tiles each, with *G. barbata* recruits, were placed in front of the Marine Biology Station Piran, at 2.7 m of depth. The sea bottom of this site used to be completely natural, composed by sandstone boulders, until 2016, when pebbles were placed in the depth range from 0 to 2 m during the construction of a beach. The lower infralittoral and the circalittoral, characterized by a gentle slope, are mainly sandy, therefore the site is influenced by a moderate resuspension of sediments. On the day of deployment the salinity was around 36, and the temperature 18.5 °C. The tiles were fixed on the plates (with screws) within an hour, while the plates were placed in tanks with seawater. After fixing, the tanks were immediately transported to the shore and then the sea bottom within 2 h.

Photographic monitoring of the plates started after one month in the sea, on 16 June (Time 1, Figure 3G), and continued over the following three months: on 17 July (Time 2, Figure 3H), 19 August (Time 3, Figure 3I), and 30 September (Time 4).

On 14 August, the Slovenian coast was hit by a storm, a strong tramontane wind that generated large waves. During this meteorological event, the cage protecting plate A was removed and it was not found during SCUBA surveys conducted on the following days. Therefore, it was necessary to build a new cage that was placed on plate A on 19 August.

**Figure 6:**
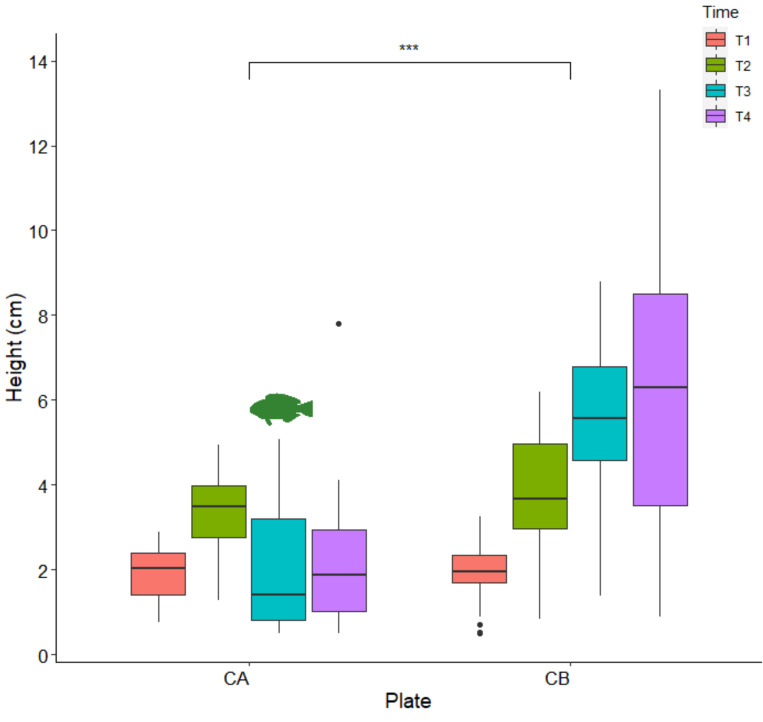
Length of juveniles on the two plates (CA and CB) from Time 1 to Time 4. The fish icon indicates the period during which plate A remained unprotected for 5 days (*n* = 30), coloured box represents interquartile range, central bar represents the median, vertical lines indicate the largest values within 1.5 times the interquartile range above the 75° percentile or the smallest below the 25° percentile, black points are values more than 1.5 times and less than 3 times the interquartile range. *** *p* < 0.001 (2-ways robust ANOVA).

The growth of outplanted juveniles differed significantly between the two plates after the loss of the protection cage of plate A (Figure 6, Robust two-ways ANOVA *p* < 0.0001, Table 1). In fact the average height of juveniles did not differ between plates at Time 1 (plate A = 1.9 cm ± 0.09 SE; plate B = 1.9 cm ± 0.07 SE) and Time 2 (average height plate A = 3.3 ± 0.16 SE plate B = 3.6 ± 0.31 SE) (Tukey test, *p* > 0.05). Conversely, after the loss of the first protection cage, at Times 3 and 4, the average height of juveniles was lower on plate A (Time 3 = 2.0 cm ± 0.28 SE; Time 4 = 2.2 cm ± 0.29 SE) than on plate B (Time 3 = 5.6 ± 0.42 SE; Time 4 = 6.2 ± 0.56 SE) (Figure 6, Tukey test, *p* < 0.05).

**Figure 7:**
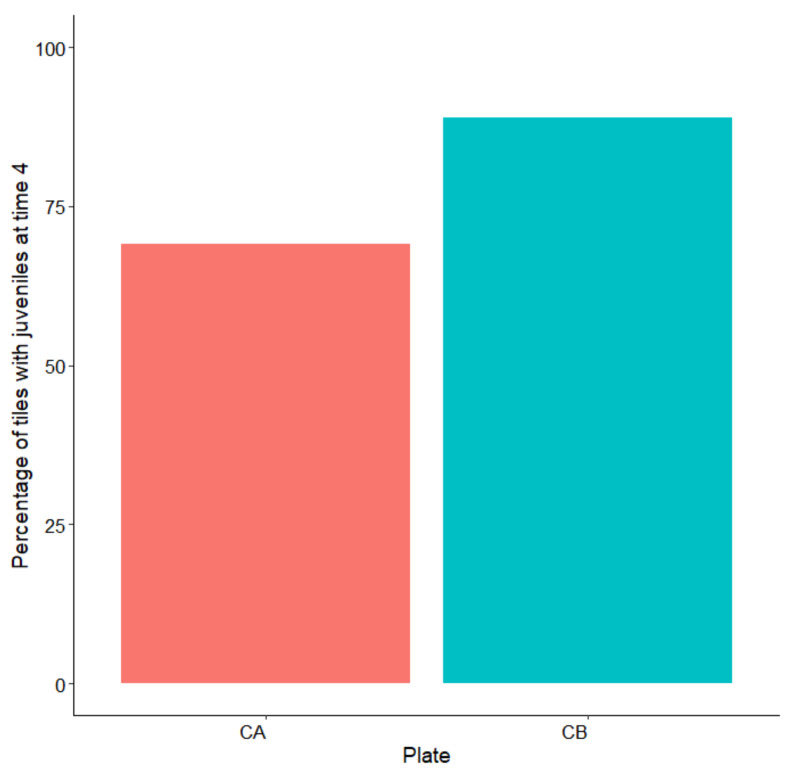
Percentage of tiles with juveniles after four months (Time 4) on the two plates (CA and CB).

The survival of the thalli was assessed on 30 September. After four months in the field (Time 4), juveniles were present on 89% of the tiles on plate B, and on 69% of the tiles on plate A (Figure 7).

## 4. Discussion

The ongoing decline of brown algal forests along Mediterranean rocky shores is causing an exceptional biodiversity loss [18,21]. Although coastal urbanization (triggering habitat damage), nutrient enrichment and chemical pollution have been recognized among the major stressors leading to the disappearance of vulnerable canopy-forming species [18,29,58], in some areas, such as in the northern Adriatic Sea, recent studies underline that eutrophication has decreased substantially [60,67]. Therefore, other acting factors acting in synergy should be considered in the evaluation of benthic habitat changes, especially in the view of habitat restoration. Due to their limited dispersal ability, when *Cystoseira* species disappear from large geographical areas, their populations can recover significantly only by restoration activities that increase recruitment [43,44].

Despite several experimental constrains, the first restoration experiment for *G. barbata* in Slovenian coastal waters produced promising results. Comparing them with those of Savonitto et al. [45] for the Italian part of the Gulf of Trieste, some important conclusions are drawn. Firstly, not only increasing winter temperatures in the Gulf, as reported for 2019 by Bevilacqua et al. [68] and Savonitto et al. [45], but also exceptional wind periods (like in spring 2020, Figure 2), lead to serious biological anomalies and the loss of the reproductive potential of *G. barbata*. In both cases, the receptacles collected were smaller than those that usually mature in spring on primary branches, and they were even growing on adventitious branches in February 2019. Moreover, during our experiment, a significant delay was observed in the release of zygotes and the occurrence of the first mitoses compared with the results of Savonitto et al. [45], which leads us to assume that the eggs had finished maturing in the conceptacles while they were already in culture. Our current knowledge on such anomalies on adult thalli in a restoration context is very limited [45], but other negative effects of increasing temperatures on algal forests have already been reported [69,70,71,72]. Moreover, as regards the nearest geographical area, the western Croatian Istrian coast, Iveša [59] hypothesized a decisive role of high summer temperatures on a large regression of *Cystoseira* canopies, even at sites that are not directly threatened by anthropogenic pressures. In any case, the interaction between different stressors remains largely unexplored, thus limiting the capacity of researchers to predict the effects of climate change [73]. The seasonality of algal reproduction is driven by environmental signals acting on different time scales, and changes in regional wind patterns and sea surface temperatures can become problematic for marine sexual reproduction [74], leading to severe ecological consequences. In the current framework of climate change, since predictions of weather and climate are inherently uncertain, the reproductive stochasticity can pose a major risk for the restoration of brown algal forests [45]. Therefore, monitoring the phenological changes of *Cystoseira* thalli is not sufficient; future research on thermal responses of canopy-forming species should be planned to facilitate restoration actions. Olischläger and Wild [74] consider that another silent threat requires experimental evaluation, namely, the possible impact of ongoing ocean acidification on the lower spawning of some important macroalgal species. This process could potentially favor opportunistic species, which are able to grow faster by applying vegetative reproduction strategies. In any case, seawater warming and other climate-related pressures will not be mitigated over short time scales, and some scientists are already discussing the potential applications of synthetic biology to algal forest conservation, either directly by manipulating algal genomes, or indirectly using approaches that do not involve the algae themselves, such as engineering associated microbiomes [75].

Due to delayed zygote release during laboratory cultivation, we were forced to leave the apices in the aquaria for almost four days and to perform the first water change on day 8, when zygotes were well-attached to the tiles. This delay, compared with the finding of Savonitto et al. [45], caused consistent growth of bacteria and other epiphytes. Moreover, the germlings of *G. barbata* showed slow growth (Figure 3 and Figure 5), slower than the growth reported for the second cultivation effort of Savonitto et al. [45]. Furthermore, due to severe biofilm problems, the young thalli were transferred to the sea much earlier than planned, when they were still very small (just over 1 mm on average). Therefore, it was not expected that they would grow as fast in the natural environment (Figure 6). In fact, after about four months in the sea, on the constantly protected plate B, the average height of the juveniles was 6.2 ± 0.56 SE (Figure 6), while during the first cultivation of Savonitto et al. [45], this height was achieved just after six months (6.2 cm ± 0.4 SE) in the protected treatment. Moreover, after four months in the field, our young *G. barbata* thalli were present on 89% of the tiles on plate B and on 69% of the tiles on plate A (Figure 7), while in the protected treatment of the first culture of Savonitto et al. [45] after six months, juveniles were present only on 46% of the tiles. Based on these results, we can assume that the site in front of the Marine Biology Station in Piran, where the two concrete plates with recruits of *G. barbata* were placed, is more suitable than the WWF Marine Protected Area of Miramare in the Italian part of the Gulf of Trieste, where the tiles of Savonitto et al. [45] were placed. In fact, less than a decade ago, a dense brown algal forest was present in front of our Institute, composed mainly by *G. barbata*, *C. compressa*, *Padina pavonica* (L.) Thivy, and other smaller algal species [56], which was completely destroyed in 2016, due to invasive interventions for the construction of the beach, when no precautions were taken to limit environmental damage. The two sites in the Gulf of Trieste do not differ significantly in terms of substrata and exposure to winds and currents, since both can be defined as “rocky shallow moderately exposed” [56]. However, the Miramare MPA is located in an area that is subject to high nutrient availability in the spring and autumn periods due to riverine discharges, especially from the Isonzo River [76], while the Piran site is influenced mainly by smaller rivers [77]. Thus, we can assume that it is less eutrophic in general, even if nutrient data are not collected from such shallow waters, but at a distance of 1.3 km from the coast [77]. According to the TRIX index, that represents a combination of pressure (Dissolved Inorganic Nitrogen and Total Phosphorus) and impact indicators (chlorophyll a and oxygen absolute deviation from saturation), the water quality conditions of Slovenian coastal waters was evaluated as Elevated (oligotrophy) [78]. Moreover, the main pressure exerted on unprotected young *G. barbata* thalli, once placed in the natural environment of the Miramare MPA, was grazing by native herbivorous species, such as *Sarpa salpa* (Linnaeus, 1758), as reported by Savonitto et al. [45]. Other recent studies have concluded that grazing by both native and invasive fish represents a silent threat for brown algal forests in temperate areas [6,41,79,80]. Moreover, according to Gianni et al. [40], *S. salpa* is able to eliminate up to 90% of transplanted thalli in a few days. During our summer monitoring surveys of the concrete plates with young *G. barbata* thalli, we also observed many specimens of *S. salpa* feeding on the biofilm developed on the cages. Therefore, we assume that it was this native species that fed on the thalli growing on plate A, which remained unprotected during five days in August 2020. In the last twenty years, the sea urchin *Paracentrotus lividus* (Lamarck, 1816) posed no threat to brown algal forests (*pers. obs*.), while in the 1970s, a large part of the macroalgal cover along the Slovenian rocky infralittoral belt almost disappeared due to overgrazing by this species [81]. Macroalgal communities started to reappear in the coastal area from 1992 [82].

Since it is able to drastically reduce algal and seagrass canopies, *S. salpa* has been defined as an important ecosystem modifier [83]. Currently, fluctuations in herbivore populations cannot be easily controlled, so they are considered a big obstacle to ecological restoration. Therefore, grazing is one of the factors that should be kept under control during the growth of canopy-forming thalli. In order to protect young thalli from grazers, different innovative techniques have been developed and tested, including the fish deterrent device DeFish [6], and anti-grazing devices consisting of flexible metallic mesh strips placed around each tile with germlings [45].

In conclusion, the results of this study confirm that outplanting infralittoral canopy-forming species, like *G*. *barbata*, is an operable approach for restoration efforts in the northern Adriatic Sea. Our first experiment in Slovenian coastal waters provides new information for the optimization of the best practices during the laboratory cultivation and addresses the early steps of restoration and introduction of young thalli in the natural environment. Our findings stress that during the laboratory culturing phase the use of an appropriate antibacterial solution in addition to the culture medium is highly recommended to guarantee high densities of healthy embryos and increase the potential for restoration success. Due to the lack of antibiotics, we had to renew the culture medium every day and continuously aerate the aquaria in order to make the thalli grow to the minimum height necessary for their outplanting, which was not to be taken for granted, given the complete coverage of the tiles by the biofilm. However, the positive result of the laboratory phase does not yet constitute a guarantee for the success of the outplanting in the coastal sea. Even in geographical areas where high levels of environmental quality have been achieved after restoration measures, the successful reintroduction of *Cystoseira* species is biased by other factors, some uncontrollable, like increasing sea temperature [84] and exceptional meteorological events, and some more predictable, such as pressure due to herbivorous fish assemblages. With that in mind, even if in situ techniques were cheaper, since they require less infrastructure and maintenance [44], the ex situ cultivation technique offers some advantages for reforestation actions, such as the decrease of high mortality rates experienced by the early life stages of *Cystoseira* spp. in the sea [85]. Prolonged periods of culture can mitigate this problem, and thus ensure more successful restoration actions [44]. Given the knowledge gap in the field of brown algal forests restoration, more experiments and studies focused on the dispersal ability of *Cystoseira* spp., and the ecology of their recruits and juveniles are required, before planning large-scale sustainable reforestation actions.

## Figures and Tables

**Table 1:** Two-ways robust ANOVA with repeated measures.

Factor	*df*	Sum sq	Mean sq	F-Ratio	*p*-Value
Time	3	303.9	101.3	39.8	<0.0001
Plate	1	138.7	138.7	54.5	<0.0001
Time × Plate	3	215.5	71.8	28.2	<0.0001

*df* = degrees of freedom, Sum sq = sum of squares, Mean sq =mean squares.

## Data Availability

The data presented in this study are available on request from the corresponding author.
